# What Works for Whom: Patients' Psychological Resources and Vulnerabilities as Common and Specific Predictors of Working Alliance in Different Psychotherapies

**DOI:** 10.3389/fpsyt.2022.848408

**Published:** 2022-07-05

**Authors:** Erkki Heinonen, Paul Knekt, Olavi Lindfors

**Affiliations:** ^1^Department of Psychology, University of Oslo, Oslo, Norway; ^2^Department of Public Health and Welfare, Finnish Institute for Health and Welfare, Helsinki, Finland; ^3^Department of Psychology and Logopedics, University of Helsinki, Helsinki, Finland

**Keywords:** patient characteristics, psychotherapy process, depression, anxiety disorders, precision medicine, brief psychotherapies, long-term psychotherapy

## Abstract

**Background:**

Across different types of psychotherapy, one of the most robust predictors of better therapeutic outcomes is a good working alliance between patient and therapist. Yet there is little comparative research on whether particular patients more likely achieve a better alliance in certain treatments which represent particular therapeutic approaches or durations.

**Methods:**

326 patients suffering from depressive and/or anxiety disorder were randomized into two short-term (solution-focused or psychodynamic) and one long-term (psychodynamic) therapy models. Treatments lasted ~7 and 36 months, respectively. Before randomization, patients were assessed with the interview-based Suitability for Psychotherapy Scale and filled Childhood Family Atmosphere and Life Orientation Test questionnaires. Patients filled Working Alliance Inventory after 3rd therapy session and at end of treatment; the long-term therapy patients, additionally, at 7 months' time point. Linear regression models were used.

**Results:**

Greater psychological resources (e.g., capacity for self-reflection, affect regulation, flexible interaction) had little effect on alliance during the course of the short-term therapies. However, they did predict better working alliances at end of long-term as opposed to short-term therapy. Childhood adversities impacted alliances already at 7 months.

**Conclusions:**

Although patients with certain qualities achieve better alliances in long-term as opposed to short-term therapies, apparently the theoretical orientation of therapy makes little difference. For patients with childhood adversities, differences between long-term (psychodynamic) treatment vs. various brief therapy models may be particularly salient.

## Introduction

When looking at average outcome, various psychotherapy models appear equally effective for common mental disorders, such as depression and anxiety (1, 2). Yet the outcomes vary notably between patients, with many individuals not achieving clinically significant change and 5–10% even deteriorating (3). Would some of these patients have recovered more likely in another type of therapy than the one they received?

Addressing this question is the goal of individualized or precision psychiatry. The pertinent question is: Which treatment, psychological or pharmacological, works best for a patient with given characteristics, over and above diagnosis (4)? Several challenges are involved in this question. Firstly, depressive and anxiety disorders are multifaceted syndromes with numerous possible predisposing, precipitating, perpetuating, and protective factors. These factors range from genetic influences to facets of personality (e.g., liability to negative and positive affect) to early life events (e.g., childhood adversities) and current circumstances (e.g., interpersonal relationships)—many of which treatments might address (5–8). Secondly, there is still little shared understanding of how psychotherapy or its different forms actually work (9).

Despite this complexity, increasing research interest and recent statistical applications, such as the Personalized Advantage Index (PAI), yield lists of patient pre-treatment variables which may favor a certain type of psychotherapy over another for better outcomes (4, 10). However, these clinical support tools would ideally be complemented by some understanding of *why and how* particular patient pre-treatment characteristics matter (11). To this end, more research is indicated also on how effective therapy processes are facilitated or not by patients' pre-treatment qualities. Moreover, such investigations should be carried out across and between different therapy models, which is the aim of the present study.

Across different types of therapy, one of the most robust and consistent predictors of better therapeutic outcomes is the therapeutic relationship or *working alliance* (12). The alliance is typically conceptualized to involve agreement on treatment tasks and goals as well as a mutual sense of liking and trust between patient and therapist (12, 13). Significantly, the alliance appears to have both between- and within-patient effects on outcome (14). This means, firstly, that patients with generally stronger alliances improve more than patients with generally weaker alliances. Secondly, it means that if a given patient's alliance improves during therapy, he or she will likely have a better outcome. Both observations, therefore, suggest that treatment outcomes might plausibly be improved by guiding patients into the types of psychotherapy where they are most likely to form strong alliances: either over the course of therapy or, ideally, right from the start of therapy.

Yet little is known of, firstly, whether certain patient qualities facilitate alliances across all or most forms of therapy, and, secondly, whether others predict a better fit with the goals and tasks of a particular treatment. In other words, more knowledge is needed on patients' pre-treatment characteristics that serve, as we term them, as common and specific predictors of working alliance in different kinds of therapy models. “Common” predictors would be characteristics expected to facilitate a good working alliance in all sorts of psychotherapy. These would likely encompass qualities such as motivation for treatment (15), self-reflective capacity (16, 17), flexibility in interaction (18, 19), and the ability to experience, express, and regulate affect (20, 21). The extant research on such characteristics has nevertheless tended to focus on relatively treatment-resistant, long-term problems, such as substance abuse, eating, personality, and psychotic disorders (20, 21). Further, their impact has rarely been compared in different treatment types. Nevertheless, while such evidence is still lacking, these qualities could plausibly be expected to facilitate therapeutic work also in depression and anxiety and across different therapy models.

“Specific” predictors, in turn, would be those indicating a good “fit” with a particular therapy approach. Such pre-treatment predictors might comprise, for instance, various cognitive, emotional, and relational styles of patients that fit with the goals and tasks of a particular treatment model. The little extant research offers some promise that such associations can be identified. An example would be the plausible finding that a patient's interpersonal rigidity may be more detrimental to working alliances in a more directive approach, such as a cognitive-behavioral one, than a less directive approach, such as an interpersonal one (22). Another quite plausible (23–25), but virtually uninvestigated characteristic is the patient's understanding of the etiology of his or her problems, so that it aligns with the conceptual model of the therapist. For example, psychodynamic treatment models typically see childhood as important for current distress and work with it in various ways, e.g., through transference and interpretation. Consequently, a strong alliance formed via a shared understanding of tasks and goals, specifically in psychodynamic treatment, might be predicted by a patient's subjectively felt unhappy childhood or a positive reaction to a clinical interpretation of his or her recurring problems that could be jointly worked on Malan (26). Such a hypothesis is supported by both qualitative (25) and quantitative (27) outcome studies. These studies have indicated that if patients recall an unhappy childhood and see it as central to their current difficulties, psychodynamic treatment can be a particularly viable approach.

In contrast to psychodynamic therapy, solution-focused therapy puts explicitly little emphasis on the past. Instead, it emphasizes constructing solutions and positive visions of the future, building on the patient's resources. Accordingly, while an optimistic disposition, i.e., an assumption that things work out somehow (28),—is a resource that might be beneficial in many kinds of personal troubles as well as treatments (29), it might be especially useful for engaging in the techniques of solution-focused therapy, e.g., imagining scenarios where one's problems have been solved overnight and how that would further positively impact one's behavior (30, 31). However, despite the plausibility of these hypothesized “specific” predictors, we are not aware of prior research tested these assumptions in a comparative design.

Finally, there are characteristics which might hinder developing the alliance in almost all types of therapies. For instance, a lack of clear treatment focus in the patient's problem would be expected to challenge joint formulation of treatment goals and tasks, no matter what type of therapy. Likewise, severe childhood abuse (32) or an overly punitive or unrealistic self-concept (33) might be expected to hinder the formation of a trusting relationship as the basis of the therapeutic bond. Circumstantial evidence exists for such assumptions. For instance, patients with complex problems take more time in therapy to recover (34). Further, those abused during childhood have been observed to stay longer in treatment and improve more slowly (35). Finally, patients with borderline features, characterized by harsh and idealized self- and other-representations, often have more trouble establishing positive therapeutic alliances with their therapists (36). Yet many authors and clinicians consider that even with these challenges, issues of trust and bonding can be worked around to lead to better alliances, especially during a long-term treatment (34). However, the above literature has tended to focus on outcome research. No research to our knowledge has compared whether characteristics such as mentioned above are predictive of a better alliance at the end of long-term therapy as opposed to short-term therapy, when treating depressive and anxiety disorders.

The Helsinki Psychotherapy Study is a randomized clinical trial of the effectiveness of two short-term (psychodynamic and solution-focused) psychotherapies and one long-term (psychodynamic) psychotherapy in the treatment of depressive and anxiety disorders. In order to better understand how the alliance forms and develops in these three treatments of different theoretical approaches and durations, we sought to test the abovementioned hypotheses, as summarized below:

*Hypothesis 1: Common predictors of working alliance*.

Better motivation for treatment, capacity for self-reflection, flexibility in interaction, and ability to regulate affect will predict better working alliances in all three types and durations of psychotherapy.

*Hypothesis 2: Orientation-specific predictors of working alliance*.

*Hypothesis 2.1:* A subjectively felt unhappy childhood and a positive reaction to a clinician's trial interpretation of the patient's central repeating conflict or problem area will predict a better alliance both within short- and long-term psychodynamic therapies and as compared to the alliance in solution-focused therapy.

*Hypothesis 2.2:* An optimistic personality disposition will predict a better alliance both within solution-focused therapy and as compared to the alliance in short- and long-term psychodynamic therapy.

*Hypothesis 3: Duration-specific predictors of working alliance*.

Poor treatment focus, poor self-concept, and physical or sexual abuse during early childhood will predict a better alliance at the end of long-term (psychodynamic) therapy as compared to the end of short-term (psychodynamic and solution-focused) therapy.

## Materials and Methods

The Helsinki Psychotherapy Study (HPS) was initiated in 1994 to study the effectiveness of three forms of psychotherapy—solution-focused therapy, and short- and long-term psychodynamic therapy—in the treatment of depressive and anxiety disorders. More comprehensive details on the design and participants have been published (37, 38).

### Patients

A total of 326 psychiatric outpatients, aged 20–46 years, Caucasian, and suffering from depressive and anxiety disorders, were recruited from the Helsinki region and randomized into solution-focused, short-term psychodynamic, or long-term psychodynamic psychotherapy. Patients eligible for the study had a long-standing (>1 year) disorder causing work dysfunction and met DSM-IV criteria for anxiety or mood disorder (39). Exclusion criteria were bipolar I, psychotic, severe personality, adjustment, substance abuse, or organic disorder. Individuals also excluded comprised those treated with psychotherapy within the previous 2 years, psychiatric health employees, and persons known to the research team members.

### Therapies

Solution-focused therapy (SFT) is a brief, resource-oriented, goal-focused therapeutic approach that helps clients change by constructing solutions (31), as developed and described by de Shazer et al. (30). Short-term psychodynamic psychotherapy (SPP) is a brief, focal, transference-based therapeutic approach that helps patients by exploring and working through specific intrapsychic and interpersonal conflicts, as described by Malan and Sifneos (26, 40). Long-term psychodynamic therapy (LPP) is an open-ended, intensive, transference-based therapeutic approach that helps patients by exploring and working through a broad range of intrapsychic and interpersonal conflicts, following the clinical principles of LPP (41).

The frequency of sessions in SFT was flexible, usually one session every 2–3 weeks, up to a maximum of 12 sessions, over no more than 8 months. SPP was scheduled for 20 treatment sessions, with one session per week. The frequency of sessions in LPP was 2–3 times a week for ~3 years.

SFT was manualized. Adherence was monitored by means of clinical supervision and feedback which were provided throughout treatment, partially online. The supervision and feedback focused on ensuring the therapist defined concrete treatment goals collaboratively with the patient; kept a positive and resource-oriented stance; and employed solution-focused techniques to foster competence and positive visions of the future (42).

Psychodynamic psychotherapies were conducted as in standard clinical practice, where the therapists might modify their interventions according to the patient's needs within the respective framework. Therefore, no manuals were used and no adherence monitoring was organized. For all treatments, external criteria on the provision of therapy according to protocol (i.e., number and frequency of sessions, possible interruptions, and duration of therapy) were monitored.

### Therapists

A total of 55 therapists provided the treatments (43). SFT was provided by 6, SPP by 12, and LPP by 41 clinicians overall, with 4 therapists providing both SPP and LPP. A local institute had trained and ensured the qualifications of all therapists providing SFT. One of the accredited Finnish psychodynamic or psychoanalytic training institutes had trained and ensured the qualifications of all therapists providing SPP or LPP. In addition, those giving SPP had received 1–2 years of specific short-term focal psychodynamic therapy training. Therapists providing SFT had on average 9 years of work experience, therapists providing SPP had 16 years, and those providing LPP had 18 years.

### Measures

#### Suitability for Psychotherapy Scale

The majority of the patient predictors were assessed with the interview-based Suitability for Psychotherapy Scale (SPS) (44). The SPS was evaluated at baseline, before initiating the treatments, in interviews conducted by trained and experienced clinicians who were not involved in the patients' treatment (44). The clinician-rated SPS is intended to assess capacities for psychotherapeutic work and comprises seven domains: motivation for treatment, self-reflective ability, flexibility in interaction, capacity for affect regulation (“modulation of affects”), reaction to a trial interpretation made by the clinician, stability and coherence of self-concept (“self-concept in relation to ego ideal”) and the degree to which a patient's problems may be conceptualized as having a circumscribed, clearly identifiable focus (44).

These seven domains were assessed for each patient over the course of three initial interview sessions. In the interview, the interviewer presented several opening questions for the patient regarding the predominant complaints that had made him or her seek therapy. After that, the interviewer focused on observing the patient's capacity to elaborate on the theme, offering clarifications and making confrontations if necessary, and evaluating the patient's ability to utilize them; as well as evaluating the here-and-now affective and reflective functioning and identity issues of the patient, as well as the interaction with the interviewer. The domains were rated on a 1–7 ordinal scale, lower values indicating higher suitability.

Based on a priori conceptual and clinical considerations, derived from the work of international pioneers of psychotherapy suitability for short-term psychodynamic psychotherapy (26, 40), the ratings were grouped into three classes, of “low”, “intermediate” or “high” level. For affect regulation, for instance, the “low” class was characterized by good ability to access positive and negative affect or, at worst, mild defensiveness; “intermediate” by somewhat restricted contact with affects, but having no major impact on the interview; and “high” group ranging from significant defensiveness that restricted the interview to affective outbursts, stupor, or agitation. [For further details on rating each individual domain, please see (44)]. For greater statistical power, these were then combined into two groups, merging the “intermediate” group either with the “low” or “high” group, so that the two groups were clinically meaningful and as equal in size as possible. The resulting “good” and “poor” categories of suitability have been found reliable and valid for predicting outcome in short- and long-term therapies that represent different theoretical orientations (45, 46).

#### The Childhood Family Atmosphere Questionnaire

The Childhood Family Atmosphere Questionnaire (CFAQ), filled at the baseline, is a 17-item self-report questionnaire for adults, informed by family, trauma, and attachment research (47). It was developed in the HPS to assess the significance of childhood adversities when planning for psychotherapy. It has three scales soliciting information on childhood family unhappiness (positive vs. negative experiences of parental care, family relations, and overall atmosphere), parental problems (marital, mental, somatic, economic, alcohol-related), and abuse (both sexual and physical) before 8 years of age. The respective scale scores are calculated as the average of the items (rated on a scale from 1 = “I totally agree” to 5 = “I definitely do not agree”), some of which are reverse-scored so that the direction for negative and positive ratings is the same. The scales have demonstrated reliability; concurrent validity (with associations to generally poorer psychological functioning); and being predictive of psychotherapy outcome (27, 47). In the current study, given our hypotheses, only the scales for childhood family unhappiness and abuse were investigated.

#### Dispositional Optimism

Dispositional optimism was rated by patients at the baseline with the Finnish version (48) of the Life Orientation Test questionnaire (LOT) (28). The questionnaire consists of eight items, four of which are keyed in a negative direction (e.g., “I hardly ever expect things to go my way”) and four of which are keyed in a positive direction (e.g., “I always look on the bright side of things”), rated on a scale from 0 = “Strongly disagree” to 4 = “Strongly agree”. In addition, the questionnaire has four filler items (e.g., “I don't get upset too easily”). Dispositional optimism is calculated as the direct sum score of the items, after reversing the negatively-keyed items.

#### Other Baseline Measures

Patients rated their current psychopathological symptoms at baseline with the Symptom Checklist 90 self-report, which yields scores for overall symptoms (SCL-90-GSI), depression (SCL-90-DEP), and anxiety (SCL-90-ANX) (49), and Beck Depression Inventory (BDI) (50). Psychiatric diagnoses on Axes I and II were assessed by experienced clinicians with DSM-IV criteria using a semi-structured interview (39). Also, they rated the patients' depressive symptoms with the Hamilton Depression Rating Scale (HDRS) (51), anxiety with the Hamilton Anxiety Rating Scale (HARS) (52), and the patient's general level of functioning with the General Assessment of Functioning scale (GAF) (39).

Information on earlier episodes of major depressive disorder, onset of the first psychiatric disorder, duration of primary psychiatric disorder, suicide attempts, previous psychotherapy and psychotropic medication was solicited with questionnaires developed in the trial. Further data on patients' sociodemographic factors (sex, age, marital status, education) were likewise assessed with questionnaires (38).

#### Outcome Measure

Working alliance was assessed by the patient-rated version of the Working Alliance Inventory (WAI) (53). WAI consists of 36 items, a third of which each measure agreement on (i) the goals of treatment; (ii) the agreement on the therapeutic tasks; and (ii) the affective bond between patient and therapist. The total score was calculated by first adding up the scores of all items (rated on a 7-point ordinal scale, from 1 = “never” to 7 = “always”), after reversing 14 negatively worded items. The WAI was rated by all patients after the third therapy session and at the 7 months' measurement point after the initiation of treatment, when the short-term therapies were ending. In addition, it was rated by the patients randomized into long-term therapy at the 36 months' measurement point, when the long-term therapies were ending.

### Statistical Methods

A cohort study design with repeated measurements was used. All the patients who had been randomized and participated in the measurement points were included in the analyses. The analyses were based on the assumption of ignorable dropouts during follow-up (54). The model for the dependent variable was a linear mixed model with an individual linear random effect component (55). Random effects were assumed to follow a normal distribution with zero means. The repeated measurements were assumed to be independent given the random effects, baseline covariates, and model parameters. The baseline covariates were modeled as fixed effects (54).

The dependent variable was the working alliance (WAI). The independent variables of the model included the patient pre-treatment predictor measured at baseline (i.e., SPS, CFAQ, and LOT, one at a time), therapy group, and the time of measurement during follow-up. Further, they included these variables' first- and second-order interactions, and nine factors satisfying criteria for confounding (56), i.e., sex, age, marital status, diagnosis, duration of primary mental disorder, previous therapy, patient-rated depressive symptoms (BDI), interview-based depressive symptoms (HDRS), and interview-based anxiety symptoms (HARS). The model further included variables on deviations from the standard treatment protocol, i.e., waiting time from randomization to the initiation of treatment, the difference between theoretical and realized date of measurement, which was a correction term, applied due to the assessments not occurring exactly at the same time for all participants (e.g., some patients answering questionnaires only after a reminder), and withdrawal after randomization, measured at baseline, and the time-dependent variables on completeness of the treatment, i.e., discontinuation of treatment, the quality of the treatment, and use of auxiliary treatment (i.e., additional psychotherapy, psychotropic medication use, and hospitalization measured through the follow-up) (57).

The ordinal pre-treatment predictors were categorized to avoid potential biases resulting from the lack of information on true metric intervals between the category levels and from the linearity assumption inherent in the use of continuous variables (58). Because of the small number of individuals in the present study, the predictors, in line with prior studies, were dichotomized into two categories, “good/high” and “poor/low” values, with the SPS based on domain-specific cut-off criteria (44, 59), the CFAQ (27) and the LOT (60) by the median.

The mean differences in alliance between the therapy groups by “good/high” and “poor/low” categories of the patient pre-treatment predictor at the different measurement points were model-adjusted (61). The delta method was used for the calculation of their confidence intervals (62). The Wald test was used to test the statistical significance of the global interaction term between the therapy group, the patient pre-treatment predictor and time. It was also used to test the statistical significance of change in the alliance from the third therapy session to the 7 month measurement point for all patients, and for patients in LPP, from the 7 month to the 36 month measurement point.

All statistical analyses were performed with SAS software, version 9.3.

## Results

### Descriptive Results

The average age of the patients was 32 years, and approximately one quarter were male (Table 1). Roughly a half were single, most were working full-time or students, and every fourth had an academic degree. In all, 85 percent had a mood disorder and approximately a third of the patients had a co-morbid anxiety disorder, with a considerably smaller percentage having only an anxiety disorder and under a fifth suffering from a co-morbid personality disorder. For about a third, the primary mental disorder had lasted over 5 years, and approximately a fifth had been in psychotherapy before.

**Table 1:** Baseline characteristics of the patients by treatment group.

**Characteristic**	**Short-term psychodynamic** **psychotherapy (*N* = 101)**	**Long-term psychodynamic** **psychotherapy (*N* = 128)**	**Solution-focused** **therapy (*N* = 97)**	***p*-value** **for difference**
**Sociodemographic factors**				
Males (%)	25.7	21.1	25.8	0.63
Age (years)	32.1	31.6	33.6	0.08
Living alone (%)	48.5	49.2	56.7	0.44
Academic education (%)	19.8	28.1	28.9	0.26
Currently employed or studying (%)	85.1	75.4	83.2	0.14
**Psychiatric diagnoses**				
Mood disorder (%)	78.2	88.3	86.6	0.09
Anxiety disorder (%)	49.5	36.7	46.4	0.12
Personality disorder (%)	24.8	12.5	18.6	0.06
Psychiatric co-morbidity within Axis I or Axis II disorders (%)	48.5	36.7	45.4	0.17
**Suitability for psychotherapy scale (SPS) domains**				
Motivation for treatment (% “good”)	38.6	39.1	39.2	0.996
Self-reflective ability (% “good, incl. intermediate”)	80.2	82.8	81.4	0.88
Affect regulation (% “good, incl. intermediate”)	65.3	71.9	66.0	0.50
Flexibility in interaction (% “good, incl. intermediate”)	87.1	90.6	88.7	0.70
Reaction to trial interpretation (% “good, incl. intermediate”)	64.4	64.9	74.2	0.24
Treatment focus (% “good”)	34.0	36.7	39.2	0.75
Self-concept (% “good, incl. intermediate”)	80.2	85.2	81.4	0.59
**Other psychological characteristics**				
Childhood family unhappiness	2.77 (0.95)	2.77 (0.90)	2.86 (0.85)	0.76
Dispositional optimism	16.4 (5.51)	16.6 (5.68)	17.3 (5.62)	0.49
Childhood abuse	2.43 (1.27)	2.34 (1.05)	2.55 (1.30)	0.59
**Psychopathological symptoms and global functioning**				
*Self-rated*				
SCL-90-GSI	1.26 (0.53)	1.27 (0.55)	1.31 (0.50)	0.84
BDI	17.9 (7.53)	18.8 (8.33)	18.1 (7.76)	0.67
SCL-90-ANX	1.25 (0.66)	1.19 (0.68)	1.27 (0.72)	0.65
*Observer-rated*				
HDRS	15.4 (4.97)	15.8 (4.94)	15.8 (4.51)	0.84
HARS	15.0 (5.40)	14.8 (5.16)	14.9 (5.21)	0.96
**Psychiatric history**				
Duration of primary mental disorder over 5 years (%)	33.0	29.9	36.5	0.59
Prior psychotherapy (%)	18.8	19.0	20.0	0.98

### Predictive Results

#### Hypothesis 1: Motivation for Treatment, Self-Reflective Ability, Capacity for Affect Regulation, and Flexibility of Interaction as Common Predictors of Working Alliance Across Therapy Types

Within the three treatment types (SPP, LPP, SFT), patient-rated alliances were not predicted by the levels (“good” vs. “poor”) of the four patient characteristics (motivation for treatment, self-reflective ability, capacity for affect regulation, and flexibility of interaction) at any time point (Table 2). Between treatments, however, several statistically significant differences emerged at the end of therapies. Better alliances were rated at the end of LPP as compared to the end of SPP when patients had better motivation for treatment, interactional flexibility, affect modulation, and capacity for self-reflection (although lesser motivation for treatment also predicted the same result). In all these cases, alliances improved in LPP statistically significantly from the 7 month to the 36 month measurement point. Predictions were largely similar in the comparison of LPP to SFT, with better alliances statistically significantly predicted at the end of LPP as compared to the end of SFT by better motivation for treatment, and marginally so by better interactional flexibility (*p* = 0.07) and capacity for self-reflection (*p* = 0.06).

**Table 2:** Estimated working alliance mean values and differences in short-term and long-term psychodynamic and solution-focused therapy.

			**WAI^a^**			**WAI^a^**			
			**Mean^b^^,^^c^**			**Mean difference (95% confidence intervals)^d^**			
**Patient pre-treatment** **characteristic**	**Level of** **characteristic**	**Measurement** **point (months)**	**SPP**	**LPP**	**SFT**	**SPP-SFT**	**SPP-LPP**	**LPP-SFT**	***p* ^e^**
Motivation for psychotherapy^f^^,^^g^	“Good”	0	176.2	179.1	177.7	−1.49 (−16.5, 13.6)	−2.84 (−17.0, 11.3)	1.35 (−13.8, 16.5)	
		7	176.2	176.7	178.7	−2.49 (−18.3, 13.3)	−0.431 (−15.5, 14.6)	−2.06 (−17.6, 13.5)	
		End of therapy		192.9			**−20.1 (−35.2**, **−5.04)**	**16.7 (1.23, 32.2)**	
	“Poor”	0	176.1	170.9	179.0	−2.88 (−14.7, 8.98)	5.19 (−6.30, 16.7)	−8.07 (−20.4, 4.30)	
		7	177.7	173.4	184.5	−6.85 (−19.1, 5.42)	4.29 (−8.04, 16.6)	−11.1 (−24.0, 1.75)	
		End of therapy		188.4			**−13.9 (−26.7, −1.03)**	7.23 (−6.22, 20.7)	0.99
Self-reflective ability^f^^,^^h^	“Good”	0	175.7	172.2	177.3	−1.52 (−11.9, 8.87)	3.52 (−6.21, 13.3)	−5.04 (−15.4, 5.28)	
		7	176.8	173.1	181.3	−4.56 (−15.5, 6.37)	3.68 (−6.82, 14.2)	−8.24 (−19.0, 2.57)	
		End of therapy		189.3			**−15.6 (−26.4, −4.80)**	10.7 (−0.362, 21.8)	
	“Poor”	0	177.4	189.2	181.5	−4.15 (−26.0, 17.8)	−11.9 (−34.3, 10.6)	7.71 (−17.1, 32.6)	
		7	175.0	185.7	188.0	−13.1 (−35.8, 9.69)	−10.7 (−35.0, 13.6)	−2.36 (−28.1, 23.4)	
		End of therapy		197.9			−25.7 (−51.8, 0.504)	13.3 (−14.2, 40.8)	0.79
Capacity for affect regulation^f^^,^^h^	“Good”	0	178.1	171.4	181.9	−3.78 (−15.1, 7.55)	6.77 (−3.81, 17.3)	−10.5 (−22.0, 0.854)	
		7	178.7	172.9	185.8	−7.08 (−19.0, 4.89)	5.77 (−5.68, 17.2)	−12.8 (−24.7, −0.978)	
		End of therapy		191.5			**−16.2 (−28.0, −4.28)**	9.21 (−3.11, 21.5)	
	“Poor”	0	172.8	183.1	171.7	1.07 (−14.8, 16.9)	−10.3 (−26.8, 6.14)	11.4 (−5.86, 28.6)	
		7	174.7	179.8	176.3	−1.58 (−18.1, 15.0)	−5.03 (−22.7, 12.6)	3.46 (−14.9, 21.8)	
		End of therapy		187.5			−16.2 (−33.9, 1.49)	13.9 (−4.42, 32.3)	0.11
Flexibility in interaction^f^^,^^i^	“Good”	0	176.7	174.3	179.0	−2.38 (−12.3, 7.58)	2.39 (−7.06, 11.8)	−4.76 (−14.8, 5.31)	
		7	177.0	174.8	183.5	−6.42 (−16.9, 4.08)	2.24 (−7.94, 12.4)	−8.67 (−19.2, 1.91)	
		End of therapy		190.3			**−16.9 (−27.3, −6.42)**	9.96 (−0.889, 20.8)	
	“Poor”	0	173.4	173.3	171.5	1.89 (−26.1, 29.8)	0.062 (−30.5, 30.6)	1.83 (−32.0, 35.6)	
		7	177.5	172.7	178.5	−1.07 (−29.3, 27.2)	4.75 (−29.1, 38.6)	−5.82 (−41.4, 29.8)	
		End of therapy		207.7			−31.2 (−86.2, 23.9)	31.7 (−24.5, 87.9)	0.87
Childhood family unhappiness^j^	“High”	0	181.7	178.0	177.1	4.52 (−9.10, 18.1)	3.71 (−10.6, 18.0)	0.81 (−14.2, 15.8)	
		7	178.6	178.2	180.7	−2.08 (−16.2, 12.1)	0.426 (−15.0, 15.9)	−2.50 (−18.2, 13.2)	
		End of therapy		196.1			**−21.8 (−37.7, −5.80)**	**19.5 (3.22, 35.7)**	
	“Low”	0	173.6	168.0	182.6	−8.99 (−24.1, 6.10)	5.56 (−9.87, 21.0)	−14.5 (−31.0, 1.87)	
		7	178.2	170.7	188.6	−10.4 (−26.7, 5.88)	7.50 (−9.18, 24.2)	**−17.9 (−35.3, −0.490)**	
		End of therapy		182.7			−5.80 (−24.0, 12.4)	−3.12 (−22.2, 15.9)	0.05
Reaction to trial interpretation^f^^,^^i^	“Good”	0	176.1	172.4	178.9	−2.80 (−14.0, 8.37)	3.75 (−7.09, 14.6)	−6.55 (−17.5, 4.37)	
		7	174.1	174.1	183.3	−8.17 (−20.0, 3.62)	1.12 (−10.5, 12.8)	−9.29 (−20.8, 2.18)	
		End of therapy		190.1			**−18.4 (−30.4, −6.47)**	9.80 (−1.97, 21.6)	
	“Poor”	0	176.2	178.8	175.9	0.301 (−17.7, 18.3)	−2.57 (−18.2, 13.1)	2.87 (−16.1, 21.9)	
		7	179.0	176.4	178.6	0.431 (−18.4, 19.3)	2.62 (−14.4, 19.7)	−2.19 (−22.2, 17.8)	
		End of therapy		188.8			−12.8 (−30.1, 4.54)	13.1 (−6.96, 33.2)	0.32
Dispositional optimism^j^	“High”	0	178.1	178.4	179.3	−1.24 (−14.2, 11.7)	–.310 (−13.0, 12.4)	–.930 (−13.8, 11.9)	
		7	176.6	180.2	186.3	−9.64 (−23.5, 4.27)	−3.56 (−17.3, 10.2)	−6.08 (−19.7, 7.58)	
		End of therapy		191.8			**−18.5 (−32.4, −4.70)**	8.30 (−5.49, 22.1)	
	“Low”	0	173.9	170.4	177.6	−3.61 (−17.1, 9.86)	3.55 (−9.06, 16.2)	−7.16 (−21.1, 6.73)	
		7	177.0	169.2	179.5	−2.53 (−16.5, 11.4)	7.81 (−5.75, 21.4)	−10.3 (−24.6, 3.96)	
		End of therapy		189.0			**−15.1 (−29.2, −1.06)**	12.8 (−2.00, 27.6)	0.28
Treatment focus^f^^,^^g^	“Good”	0	178.6	177.1	182.3	−3.76 (−19.1, 11.6)	1.50 (−13.7, 16.7)	−5.26 (−20.2, 9.71)	
		7	177.4	177.3	185.8	−8.41 (−24.5, 7.71)	0.096 (−16.1, 16.3)	−8.50 (−24.1, 7.05)	
		End of therapy		190.3			−15.8 (−32.3, 0.745)	7.66 (−8.35, 23.7)	
	“Poor”	0	175.1	172.7	175.5	−0.365 (−12.2, 11.5)	2.38 (−8.70, 13.5)	−2.74 (−15.0, 9.51)	
		7	176.8	173.2	180.8	−4.03 (−16.3, 8.29)	3.54 (−8.43, 15.5)	−7.56 (−20.4, 5.24)	
		End of therapy		189.5			**−15.8 (−28.1, −3.48)**	11.6 (−1.45, 24.6)	0.97
Childhood abuse^j^	“High”	0	178.5	171.1	183.2	−4.77 (−19.0, 9.48)	7.33 (−8.16, 22.8)	−12.1 (−27.9, 3.68)	
		7	181.7	*165.2*	188.0	−6.28 (−20.8, 8.21)	**16.5 (0.378, 32.6)**	**−22.8 (−39.0, −6.57)**	
		End of therapy		182.7			−4.57 (−21.6, 12.4)	−1.55 (−18.7, 15.6)	
	“Low”	0	177.6	175.0	175.0	2.63 (−12.2, 17.4)	2.59 (−12.3, 17.4)	0.041 (−15.9, 16.0)	
		7	174.7	*184.4*	177.8	−3.16 (−18.7, 12.4)	–9.71 (−26.0, 6.60)	6.55 (−10.4, 23.5)	
		End of therapy		199.7			**−27.0 (−44.1, −9.92)**	**24.0 (6.31, 41.7)**	0.03
Stability of self–concept^f^^,^^i^	“Good”	0	174.9	173.0	176.6	−1.64 (−12.0, 8.76)	1.91 (−7.83, 11.6)	−3.55 (−13.9, 6.81)	
		7	176.2	173.2	181.2	−4.97 (−15.9, 5.91)	3.01 (−7.44, 13.5)	−7.99 (−18.9, 2.86)	
		End of therapy		187.6			**−14.5 (−25.3, −3.69)**	9.87 (−1.28, 21.1)	
	“Poor”	0	181.2	182.7	186.3	−5.17 (−26.7, 16.3)	−1.54 (−24.2, 21.1)	−3.62 (−28.0, 20.8)	
		7	180.4	184.3	189.7	−9.28 (−32.0, 13.4)	−3.97 (−29.3, 21.4)	−5.32 (−31.4, 20.7)	
		End of therapy		204.7			**−28.5 (−54.6, −2.51)**	16.7 (−9.94, 43.4)	0.66

a *Working Alliance Inventory.*

b *Statistically significant differences within a therapy group between “good/high” and “poor/low” values at a given time point in italics.*

c *Statistically significant change from the previous measurement point in underlined font.*

d *Statistically significant differences between therapy groups according to “good/high” and “poor/low” values at a given time point in bold. Point estimates of alliance differ somewhat from end-of-therapy mean difference comparisons for SPP-LPP and LPP-SFT as they were estimated from different models (mean differences having to be estimated separately from point estimates, using 7 months' time point data for SPP and SFT and 36 months' time point data for LPP).*

e *p-value for global test of interaction between patient factor, therapy group, and time throughout the follow-up.*

f *Categorized according to pre-determined criteria.*

g *Good = 1–2, Poor = 3–7.*

h *Good = 1–3, Poor = 4–7.*

i *Good = 1–4, Poor = 5–7.*

j *Categorized by the median*.

#### Hypothesis 2: Childhood Family Unhappiness, Reaction to Trial Interpretation, and Dispositional Optimism as Orientation-Specific Predictors of Working Alliance

Between treatments, patients with a happier recollection of childhood family experienced better alliances in SFT than LPP at the 7 month mark. In contrast, patients with an unhappier recollection of childhood family experienced better alliances at the end of LPP than at the end of both SPP or SFT (Table 2). The global test of interaction between therapy type, childhood family unhappiness, and time was also significant (*p* = 0.05). Further, a better reaction to trial interpretation predicted better alliance at the end of LPP as compared to the end of SPP. Both lower and higher optimism predicted a better alliance at the end of LPP as compared to the end of SPP. Within the treatment types, none of the patients' three characteristics predicted their ratings of the alliance at any time point. With regard to childhood unhappiness and dispositional optimism, alliances improved in LPP statistically significantly from the 7 to the 36 month measurement point in both “high” and “low” groups; but in the case of trial interpretation, only when the patient's reaction was “good”.

#### Hypothesis 3: Treatment Focus, Childhood Abuse, and Stability of Self-Concept as Duration-Specific Predictors of Working Alliance

Between treatments, better alliance in LPP as compared to SPP and SFT was predicted by less childhood abuse, and in LPP as compared to SPP by a less clear treatment focus and more unstable or incoherent self-concept; although a more stable self-concept also predicted the same result (Table 2). Patients with more early childhood abuse had worse alliances within LPP at 7 months into treatment (mean difference 19.2, 95% confidence interval 1.90–36.5). The global test of interaction between therapy type, childhood abuse, and time was also significant (*p* = 0.03). For all other groups except those with more abuse, alliances improved in LPP statistically significantly from the 7 month to the 36 month measurement point.

## Discussion

This study tested three hypotheses of whether and how a patient's pre-treatment characteristics predict how he or she experiences the working alliance in psychotherapy: both commonly, i.e., across three different models and lengths of therapies; and specifically, i.e., whether certain characteristics facilitate a good alliance in a particular type of treatment. Contrary to our expectations, few differences in patient-rated alliance were observed within or between therapies at the start of treatments or at the 7 months' measurement point, with respect to these common and specific patient predictors. Nevertheless, between treatments, many statistically significant associations emerged when comparing alliances at the end of the three therapies—that is, at the end of short- vs. long-term psychotherapeutic treatment. Taken together, the results show that the working alliance improves more during long- than short-term therapy; but also, that patients' pre-treatment resources and vulnerabilities may determine when such improvement is particularly notable. These findings, how they further our understanding of the alliance, and their clinical implications are discussed below.

### Hypothesis 1: Common Predictors of Better Alliances

“Common” patient characteristics expected to predict better alliances *within* all three treatment models (SFT, SPP, LPP) did not do so: not at the 3rd session nor at the respective ends of treatments. First, looking at motivation for treatment, unlike some earlier studies on similar patient problems (63, 64) and having investigated the closely-related construct of stages of change—i.e., whether patients are ambivalent about change, contemplating it, or taking action—we did not find better motivation for treatment to predict a better alliance. A possible reason is that baseline differences in motivation may have been relatively negligible in the HPS, given that all patients had signaled relatively strong motivation by consenting to a fairly arduous set of pre-treatment interviews and questionnaires that were a part of the HPS study protocol. Another reason may be that many of previous studies showing motivation to significantly predict outcomes have tended to investigate more behaviorally oriented problems, such as substance abuse and eating disorders (15), where conscious resolve for change may be central. In depression and anxiety, on the other hand, conscious personal resolve may matter less, and this reality also reflects on the alliance. In cases where patients have been suffering from long-standing depression and anxiety, such as in the HPS population, this may be especially true. The finding, in any case, underlines the need for further study on the effects of treatment motivation—especially given that significant literature shows positive treatment expectations to predict better outcomes and that such expectations can be nurtured (65).

Another surprisingly rarely studied patient predictor of alliance is a capacity for self-reflection, which also failed to have any significant within-treatment prediction on alliance. In a very rough comparison, self-reflective ability, as measured in the HPS, might be thought of as a characteristic analogous to *insight into illness* in psychotic disorders, shown to predict alliances consistently in such problems (16), i.e., as something that facilitates psychological exploration of and working with one's particular issues. Within depression and anxiety, the nearest concept may be that of mentalizing ability (66), which has produced only a few and somewhat inconsistent predictions on alliance: Taubner et al. (67) showing it to predict stronger early alliances—unlike Ekeblad et al. (17), who did not, but nevertheless saw mentalizing to predict stronger alliances across the whole course of treatment. More research is thus indicated on these kinds of self-observing characteristics and their relations to alliance.

The same conclusion holds also for the patient's capacity for affective regulation and flexible interaction. Their failure to predict alliance was noteworthy especially given that problems in these same capacities, although on a more severe level, are often seen to strain the alliance in borderline personality disorder (BPD) (36). Our findings therefore underline that caution is needed in extrapolating conclusions from one diagnostic group, such as BPD, to others, such as depression and anxiety.

Statistically significant differences were, indeed, seen only when comparing the termination points of long-term vs. short-term therapy, where better capacities predicted better alliances at the end of long-term therapy in comparison to the briefer models. In other words, patients having what appear to be good abilities for engaging in therapeutic work—i.e., motivation and capacity for self-reflection, affective regulation, and flexible interaction—were ultimately able to achieve better consensus with their therapists on treatment goals and tasks and to bond with them. Yet this effect became significantly observable only at the end of a long-term therapy process. However, it should be noted that qualities like motivation for treatment also showed statistically significant differences at the “poor” end of the spectrum, and for some other characteristics, significant differences were not far.

### Hypothesis 2: Specific Predictors of Better Alliances

“Specific” patient predictors, hypothesized to predict better patient-rated alliances in certain treatment models, by contrast to others, also did not show the expected associations. Again, significant differences were observed only when comparing long-term vs. short-term treatment. However, these findings interestingly complemented the findings from Hypothesis 1.

First, we observed working alliances to be rated as remarkably good at the end of long-term psychotherapy by those patients who felt their childhood had been relatively unhappy. It may be that those patients who felt their childhood had been unhappy particularly appreciated the chance to work through these experiences in long-term therapy—especially a psychodynamic one, which would likely address such experiences in detail. This interpretation is further supported by the fact that for those patients who felt their childhood had been *less* unhappy, better alliances were established in SFT than in LPP, and already at the 7-month mark.

The strength of this finding was further bolstered by the statistical significance of the global interaction between childhood unhappiness, treatment type, and time. The result also interestingly validates earlier qualitative findings which compared SFT and LPP using pre- and post-treatment interviews (25). In this study of “inner narratives”, those patients, who at pre-treatment saw their distress have life history-related origins (e.g., due to childhood experiences, relations with one's parents), were more satisfied and more often improved in terms of depressive symptoms at post-treatment if they had been randomized to LPP. In contrast, those patients who at pre-treatment attributed their depression or anxiety to their current life situation (e.g., present relationships or problems at work) were at post-treatment more often happy with SFT. Thus, there appeared to be a “match” between the treatment model's conceptualization of problems and the patient's own narrative about his or her problem. Accordingly, if patients were randomized to a treatment model not fitting their inner narrative, they were more likely disillusioned with and less improved by therapy (25).

A somewhat similar explanation, grounded in patients receiving treatment in line with their preferences (68), could also be given for our second finding. That is, a patient's positive reaction to pre-treatment clinical interpretation of his or her problems predicted better alliances at the end of LPP as opposed to SPP. We did not receive support for our exact original hypothesis: i.e., positive reaction to trial interpretation predicting preference of both short- and long-term psychodynamic therapy over SFT. Yet it seems plausible that those patients who responded well to a psychodynamic interpretation would appreciate a longer-term psychodynamic therapy process over a shorter one, and this could also be reflected in alliance ratings at the end of treatment.

Finally, the suitability of psychodynamic vs. solution-focused model was not supported by our findings related to the level of patients' dispositional optimism, which did not meaningfully differentiate outcomes.

### Hypothesis 3: Predictors of Better Alliances in Long-Term vs. Short-Term Therapies

Our third hypothesis focused on the differences between short- and long-term treatments, yielding partly expected and partly unexpected results. Expectedly, a poorer treatment focus predicted a better alliance at the end of LPP as opposed to short-term treatment, although only the comparison to SPP was statistically significant. However, the little differences in point estimates of “good” and “poor” categories suggest caution in making too much of this finding.

The most distinctive results were observed in relation to higher childhood physical or sexual abuse, which, at the 7 month time point, predicted significantly worse outcomes both *within* LPP and as compared to SFT and SPP. This finding is challenging to interpret without other treatment process data, such as videotapes or measurements that would indicate how these childhood issues were addressed in intensive long-term therapy. One possibility nevertheless seems that the treatment focus was on processing these traumatic experiences, perhaps unduly intensifying and complicating the therapeutic relationship relatively early in the treatment (69). It is worthwhile to note that in LPP, these patients' alliances had not improved statistically significantly by the end of treatment—whereas patients with less or no childhood abuse experienced a remarkably strong alliance at that point. The finding was further strengthened by the global test of interaction being significant (*p* = 0.03).

What are the implications of these findings for outcomes? In a prior study from the same trial, LPP patients with or without abuse history did not differ from each other on a variety of self- and clinician-rated symptomatic outcome measures, at either end of treatment or 2 years afterwards (27). Likewise, a recent comprehensive meta-analysis did not find patients with or without abuse history to differ from each other in their treatment response to pharmacological or psychotherapeutic interventions (70). The present findings bring interesting nuance to these outcome studies, suggesting that while childhood adversities do not necessarily predict worse therapeutic outcomes, they still influence patients' experience of the treatment process for better or worse in different therapeutic approaches. As such, understanding their role may require closer study, using both qualitative and quantitative methods, that may help fine-tune interventions for these patients for added benefits.

### Methodological Aspects

The strengths of the study relate to its design and comprehensive data. First, the design enabled the rare randomized comparison of therapies of both different approaches and different durations, with a relatively sizable sample. Second, the randomization appeared successful, given the lack of statistically significant differences in the baseline characteristics of patients. Third, the predictors comprised both patients' positive resources and vulnerabilities, as assessed from both their own and observers' viewpoints—thus yielding a comprehensive look into the phenomenon of alliance, as predicted by these different kinds of qualities and two subjective viewpoints. Fourth, the predictor measures have also been investigated as predictors of treatment outcome, as measured by symptomatic improvement in SPP, SFT, and LPP. This enabled evaluation of how patients' assessments of the therapeutic relationship and symptomatic outcome line up or do not line up with one another (27, 45, 46, 60). Fifth, the comprehensive data allowed for adjusting for a number of potentially confounding factors.

The study nevertheless also has several limitations. First, despite the adjustment for potential confounding factors, residual confounding cannot be fully excluded. Second, although sizable by comparison to many psychotherapy trials, the confidence intervals were large and some effects were just barely non-significant. However, given the sample size, missed effects can be assumed to be relatively subtle. Third, since therapy sessions were not recorded nor was there manualization of the psychodynamic therapies, we do not have a close understanding of what actually occurred in therapy sessions. However, this was in line with the study's intent to study standard clinical practice with ecological validity, and to be flexible according to patient needs. Fourth, more frequent assessments of the alliance would have allowed a more thorough understanding of how the alliance develops, and a more reliable assessment of alliance than one based on a couple of assessment points (71). Nevertheless, the conduct of treatments in private practice would have made significantly more frequent assessments unfeasible. Fifth, a specific limitation related to the CFAQ was that it solicited information on adverse experiences only until 8 years of age. Thus, the impact of formative adverse experiences after age 8 may have been missed by this study. Finally, also related to the CFAQ, patients' current depression and anxiety may obviously have caused recall bias of past events, i.e., priming negative recollections of childhood. However, studies have indicated that childhood adversities may be quite reliably recalled even over long periods of time (47, 72). Equally, what is currently recalled of childhood may be actually more important for therapeutic work than what “objectively” happened (20).

### Clinical Implications and Directions for Future Research

Meta-analyses have already shown that the early working alliance, assessed during the first five sessions, predicts psychotherapy outcomes relatively well, and that both patients' (*r* = 0.25) and therapists' (*r* = 0.22) assessments to be fairly equally predictive (12). Thus, apparently the patient and therapist can quickly get a telling sense of whether they are on the same page regarding the tasks and goals of therapy and connect with each other positively on an emotional level. Yet, although various patient pre-treatment characteristics would arguably be expected to help in forming this relationship, regardless of the specific therapy model and therapist—such as higher motivation for treatment, self-reflective ability, and interpersonal flexibility—their predictive power on the alliance appears virtually negligible, at least from the patient's perspective.

The lack of differences, as expected based on prior literature, may be explained by several factors. First, the design previously excluded severe personality, psychotic, and substance abuse disorders, where the associations might have been more prominent. Second, within depressive and anxiety disorders, some of the examined qualities may reflect the psychological resources of a relatively well-balanced person that shield from an overly positive or idealized view of the therapeutic relationship; but which nevertheless facilitate the patient's recovery and working through his or her problems effectively. Third, the lack of findings may underline the essentially dyadic nature of the alliance, i.e., it cannot be predicted by patient characteristics in a “vacuum”, without also taking the individual therapist into account (73).

This last reason might also explain the lack of findings regarding an optimal match between patient and therapy orientation. That is, therapists' explication of the treatment rationale and appropriate responsiveness (74) within the different therapy orientations may have enabled patients to see the value of these approaches, regardless of the patients' initial characteristics. Nevertheless, recent studies such as those applying variations of the Personalized Advantage Index suggest that treatment outcomes may be optimized by considering prescriptive patient variables that indicate suitability for a particular short-term therapy approach (10, 11). Thus, given the robust association of the working alliance to treatment outcome (12), the present findings should also be replicated before any firm conclusions are reached.

Despite the majority of our hypotheses receiving little support, some clear-cut findings in the alliance nevertheless emerged, most prominently in the case of subjectively recollected childhood adversities. As emerging meta-analytic evidence suggests childhood adversities are not an obstacle to recovery, but may sometimes even predict greater gains in pharmacological and psychotherapeutic treatment (70), the present findings should be investigated further to understand the right ways to approach and work with such adverse experiences. In other words, even if their role still needs further clarification, childhood adversities appear to be something for a clinician to be particularly aware of, and perhaps also indicate close and systematic process as well as outcome monitoring.

### Conclusions

While a good working alliance predicts better psychotherapeutic outcomes, it is harder to predict what kind of a patient will have a good working alliance. Likewise, evaluated in terms of the working alliance, patient's pre-treatment characteristics give little indication of which theoretical approach will fit them best. Nevertheless, while alliances generally improve more during long-term than short-term therapy, apparently various childhood adversities may importantly moderate the suitability of a long-term psychodynamic approach as compared to various brief therapy models. Closer research into the relationship of alliance with treatment gains and treatment satisfaction will aid understanding the clinical implications of the finding.

## Data Availability Statement

The data analyzed in this study is subject to the following licenses/restrictions: the data are not publicly available due to privacy and ethical restrictions. Requests to access these datasets should be directed to erkki.heinonen@thl.fi.

## Ethics Statement

The studies involving human participants were reviewed and approved by Ethics Council of the Helsinki University Central Hospital. The patients/participants provided their written informed consent to participate in this study.

## Author Contributions

EH wrote the original and successive drafts of the paper and had the main responsibility for the interpretation of the results. PK supervised the study, participated in all stages of the study, had the main responsibility for the statistical analyses, and commented on all drafts of the paper. OL organized the data collection, helped design the study, participated in the interpretation of the results, and commented on drafts of the paper. All authors contributed to the article and approved the submitted version.

## Funding

EH acknowledges the support of the Jalmari and Rauha Ahokas Foundation.

## Conflict of Interest

The authors declare that the research was conducted in the absence of any commercial or financial relationships that could be construed as a potential conflict of interest.

## Publisher's Note

All claims expressed in this article are solely those of the authors and do not necessarily represent those of their affiliated organizations, or those of the publisher, the editors and the reviewers. Any product that may be evaluated in this article, or claim that may be made by its manufacturer, is not guaranteed or endorsed by the publisher.
